# Evolutionary, Molecular and Genetic Analyses of Tic22 Homologues in *Arabidopsis thaliana* Chloroplasts

**DOI:** 10.1371/journal.pone.0063863

**Published:** 2013-05-13

**Authors:** Ali Reza Kasmati, Mats Töpel, Nadir Zaman Khan, Ramesh Patel, Qihua Ling, Sazzad Karim, Henrik Aronsson, Paul Jarvis

**Affiliations:** 1 University of Leicester, Department of Biology, Leicester, United Kingdom; 2 University of Gothenburg, Department of Biological and Environmental Sciences, Gothenburg, Sweden; Arizona State University, United States of America

## Abstract

The Tic22 protein was previously identified in pea as a putative component of the chloroplast protein import apparatus. It is a peripheral protein of the inner envelope membrane, residing in the intermembrane space. In Arabidopsis, there are two Tic22 homologues, termed atTic22-III and atTic22-IV, both of which are predicted to localize in chloroplasts. These two proteins defined clades that are conserved in all land plants, which appear to have evolved at a similar rates since their separation >400 million years ago, suggesting functional conservation. The *atTIC22-IV* gene was expressed several-fold more highly than *atTIC22-III*, but the genes exhibited similar expression profiles and were expressed throughout development. Knockout mutants lacking atTic22-IV were visibly normal, whereas those lacking atTic22-III exhibited moderate chlorosis. Double mutants lacking both isoforms were more strongly chlorotic, particularly during early development, but were viable and fertile. Double-mutant chloroplasts were small and under-developed relative to those in wild type, and displayed inefficient import of precursor proteins. The data indicate that the two Tic22 isoforms act redundantly in chloroplast protein import, and that their function is non-essential but nonetheless required for normal chloroplast biogenesis, particularly during early plant development.

## Introduction

The vast majority of chloroplast proteins are nucleus-encoded, synthesized on cytosolic ribosomes, and subsequently imported into plastids [1], [2], [3], [4], [5]. Most of these proteins are synthesized as precursors with N-terminal extensions called transit peptides, and are post-translationally imported into plastids after binding to the outer envelope membrane. The cleavable transit peptide is essential for chloroplast targeting and translocation across the envelope membranes. The import process is mediated by multiprotein complexes in the outer and inner envelope membranes, respectively termed TOC and TIC (Translocon at the Outer/Inner envelope membrane of Chloroplasts).

Tic22 was identified in pea as a component of the protein import machinery by its cross-linking to preproteins undergoing import across the envelope [6]. It is a hydrophilic protein with no transmembrane spans and no obvious sequence similarity to other proteins of known function [7]. It was shown to associate with the outer surface of the inner envelope membrane peripherally, as well as with other TOC and TIC components to form active supercomplexes linking the two membranes [7]. Tic22 might facilitate the passage of precursors upon their emergence from the TOC complex. In fact, it has been proposed that Tic22 acts together with other proteins, such as Toc64, Toc12 and Hsp70, to form an intermembrane space translocation complex [8].

The Tic22 protein is predicted to have a transit peptide, and deletion mutants and chimeric protein studies have shown that this is required for targeting to the intermembrane space [9]. Import of Tic22 requires ATP and protease-sensitive components on the chloroplast surface. However, competition studies revealed that Tic22 targeting to the intermembrane space does not engage the general protein import pathway used by most stromal preproteins [9]. This implied that the Tic22 presequence is not a canonical transit peptide, and that Tic22 is targeted to the intermembrane space by a novel import pathway [9]. A later study reached a different conclusion concerning the involvement of the TOC machinery, but nonetheless argued that the Tic22 presequence is processed in the intermembrane space [10].

Identification of a gene in *Synechocystis* PCC6803 with similarity to pea Tic22 indicated a cyanobacterial origin of the protein [11], [12]. More recently, a Tic22 homologue in *Anabaena* sp. PCC 7120 was studied and shown to be essential for development [13]. This protein localized in the thylakoids and the periplasm, and could be functionally replaced with a plant orthologue. Immunoprecipitation after chemical cross-linking revealed a physical interaction with the outer envelope biogenesis factor, Omp85, suggesting a function of cyanobacterial Tic22 in outer membrane biogenesis. Moreover, three-dimensional structure analysis of the *Anabaena* protein identified conserved hydrophobic pockets similar to those of ClpS or BamB, suggesting a possible chaperone function [13].

A Tic22 homologue was also identified in *Plasmodium falciparum*, and found to be peripherally associated with apicoplast membranes that are analogous to the chloroplast inner envelope membrane [14]. Subsequently, a Tic22 homologue in a similar parasite, *Toxoplasma gondii*, was shown to be apicoplast-localized and crucial for both parasite survival and protein import into the apicoplast stroma [15]. Structural analysis of *P. falciparum* Tic22 revealed a fold conserved from cyanobacteria to plants, incorporating non-polar grooves on each side of the molecule. Moreover, these grooves allow the apicoplast protein to function as a chaperone [15]. Such a chaperone had not previously been known to exist in the intermembrane space of plastids.

In Arabidopsis, two Tic22-related genes have been identified, and these are termed *atTIC22-III* and *atTIC22-IV* according to the chromosomal location of the corresponding genes [3], [16]. In this paper, we present data on the evolutionary, molecular and genetic analysis of these homologues. Very recently, another research group independently reported on the analysis of these genes [17], and so we relate our findings to those derived from that study.

## Results

### Evolution of the Tic22 Gene Family

Database searches revealed two homologues of Tic22 in Arabidopsis (atTic22-III and atTic22-IV) [16]. Full-length, sequenced cDNA clones are available for each gene (accession numbers NM_113275 and AK118805, respectively), indicating that they are both expressed in plants. Protein sequences predicted using these cDNA sequences (313 and 268 residues, respectively) were analysed *in silico*. The TargetP program predicted that both Tic22 homologues have a transit peptide (residues 1–96 and 1–59, respectively) with high confidence [18]. The corresponding mature sequences share 37.1% amino acid sequence identity with each other, and 43.2% (atTic22-III) and 73.4% (atTic22-IV) identity with the pea Tic22 mature sequence.

To begin to shed light on the functional importance of the two Arabidopsis Tic22 homologues, we analysed the evolutionary history of the gene family, by studying putative homologues from various plant and bacterial groups. Extensive searches in bacterial whole genome sequence (WGS) datasets revealed strong support for a cyanobacterial origin for the gene family. Of the 121 bacterial WGS datasets analysed, atTic22-III or atTic22-IV had a reciprocal best BLAST match in 32 of 67 (48%) cyanobacterial genomes, and only 3 of 54 (6%) genomes of other bacterial groups. Although there was a bias for cyanobacterial WGS datasets in this analysis, the different BLAST analyses nonetheless provided overwhelming support for an origin in this group. Furthermore, Tic22 sequences were found in all Archaeplastida organism groups investigated (i.e., glaucophytes, red and green algae, and land plants), implying that these proteins have an important function in the cell.

**Figure 1:**
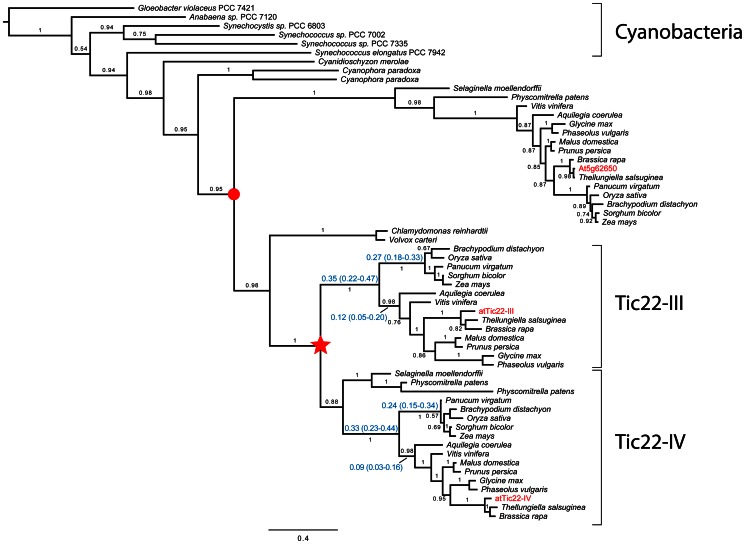
Phylogenetic analysis of the Tic22 gene family in plants and cyanobacteria. Homologous sequences identified in whole genome sequence datasets from plants and cyanobacteria were analysed using the program MrBayes. The analysis showed a strong support for an origin of Tic22 in cyanobacteria, and revealed that all investigated plant and algal species include at least one member of the gene family. A gene duplication occurred at least 416–449 million years ago (indicated by a star), and resulted in two paralogous gene copies that have been conserved in all investigated angiosperms. Branch lengths in the two clades formed by the gene duplication are very similar (mean branch length and the 95% Highest Posterior Density [HPD] credibility interval are shown for a selection of branches), which indicates that the evolutionary rates of the two paralogous copies have been similar over an extensive period of time. A partial gene duplication (indicated with a red dot) is inferred to have happened after the split between red algae and other archaeplastida. The partially-duplicated gene of unknown function has been conserved in all investigated land plant species, but has not been found in any algal species. All Arabidopsis sequences are shown in red text. Posterior probability values appear above the branches, and the expected number of changes per site along the branches is indicated by the scale bar.

Phylogenetic analysis of 58 selected Tic22-related sequences (Table S1) showed that a gene duplication happened around the time when land plants emerged, such that two paralogous gene copies are found in all investigated monocot and eudicot species (Figure 1). The two Arabidopsis genes, *atTIC22-III* and *atTIC22-IV*, derive from this event, and have hence been separated for at least 416 million years (since the split between *Selaginella* and other land plants), or as long as 449 million years (when the split between *Physcomitrella* and other land plants took place) [19]. Uncertainty in the timing of the gene duplication derives from the phylogenetic position of the clade containing sequences from *Physcomitrella patens* and *Sellaginella moellendorffii*, which is part of the Tic22-IV clade in our analysis. This result contradicts our current understanding of the evolutionary relationships amongst mosses, Lycophytes and angiosperms [20]. However, this part of the tree of life is notoriously difficult to resolve, and our result could be explained in many different ways, such as by weak or contradicting phylogenetic signals, or by deep lineage sorting (where a gene duplication happened prior to the speciation event separating mosses from vascular plants) followed by subsequent gene loss.

The lengths of selected branches following the aforementioned main gene duplication are indicated in Figure 1. These numbers show that the corresponding branches in the two Tic22 clades have a similar length, which indicates that the two copies have evolved with similar evolutionary rates. Hence, based on these results, a large functional differentiation between the two paralogous genes in Arabidopsis was not expected.

A keyword search in the Aramemnon database [21] identifies a third potential member of the Tic22 gene family in Arabidopsis (At5g62650) [17]. Attempts to include its full-length sequence in our analysis resulted in poor alignment results, as its overall similarity to atTic22-III and atTic22-IV is very low; e.g., its predicted mature domain shares just ∼21–23% amino acid sequence identity with those of the other Arabidopsis sequences or pea Tic22. Moreover, only part of this sequence (∼335 of a total of 529 residues, corresponding to exons 2–5) is homologous to the other Arabidopsis sequences (Figure S1). Putative orthologues of At5g62650 were also found in other land plant species, and the aligning regions of these were included in the analysis. Figure 1 shows that these sequences form a clade after the split between red algae and other archaeplastida, but before the split between green algae and land plants. The analysis also shows that these At5g62650-related sequences have evolved at a faster rate than the Tic22 sequences (note the longer branch lengths in Figure 1), implying that they functionally diverged during evolution in similar fashion to Toc75 and OEP80 sequences [22]. In combination, these various observations suggest that At5g62650 is not a canonical Tic22 protein and so it was not pursued further in this study.

### Analysis of Tic22 Homologue Localization

To provide experimental support for the TargetP predictions, we attempted to assess the subcellular localization of the Arabidopsis Tic22 proteins by the analysis of YFP fusion proteins. To this end, full-length coding sequences, and two truncated sequences for each one (encoding residues 1–118 and 1–273 for atTic22-III, and 1–78 and 1–224 for atTic22-IV), were inserted into the p2GWY7 vector [23] which adds a C-terminal YFP tag. However, repeated analyses of each fusion in transfected Arabidopsis protoplasts by fluorescence microscopy failed to provide clear evidence of chloroplast localization (Figure S2A; data not shown). Although some of the YFP signal may have been associated with the chloroplast envelope, the fluorescence patterns observed were generally consistent with cytosolic localization. This implies that the large YFP tag interferes with targeting to the chloroplast envelope, which might be related to the fact that Tic22 uses a different targeting pathway from most other chloroplast proteins [9], [10].

To circumvent this problem, we analysed atTic22-III as a representative protein in chloroplast import assays. Although import of atTic22-III did not result in efficient removal of the predicted transit peptide (in line with previous observations on pea Tic22 [9], [24]), post-import treatments with the proteases thermolysin (which removes proteins exposed at the outer envelope surface) and trypsin (which removes proteins in the outer envelope or others that are exposed to the intermembrane space) were consistent with atTic22-III localization in the intermembrane space (Figure S2B). These results are in agreement with those from the complementary studies of Rudolf et al. [17]. Moreover, several proteomic studies are strongly supportive of chloroplast envelope localization of both atTic22-III and atTic22-IV [25], [26], [27].

### Expression Profiles of the Arabidopsis *TIC22* Homologues

**Figure 2:**
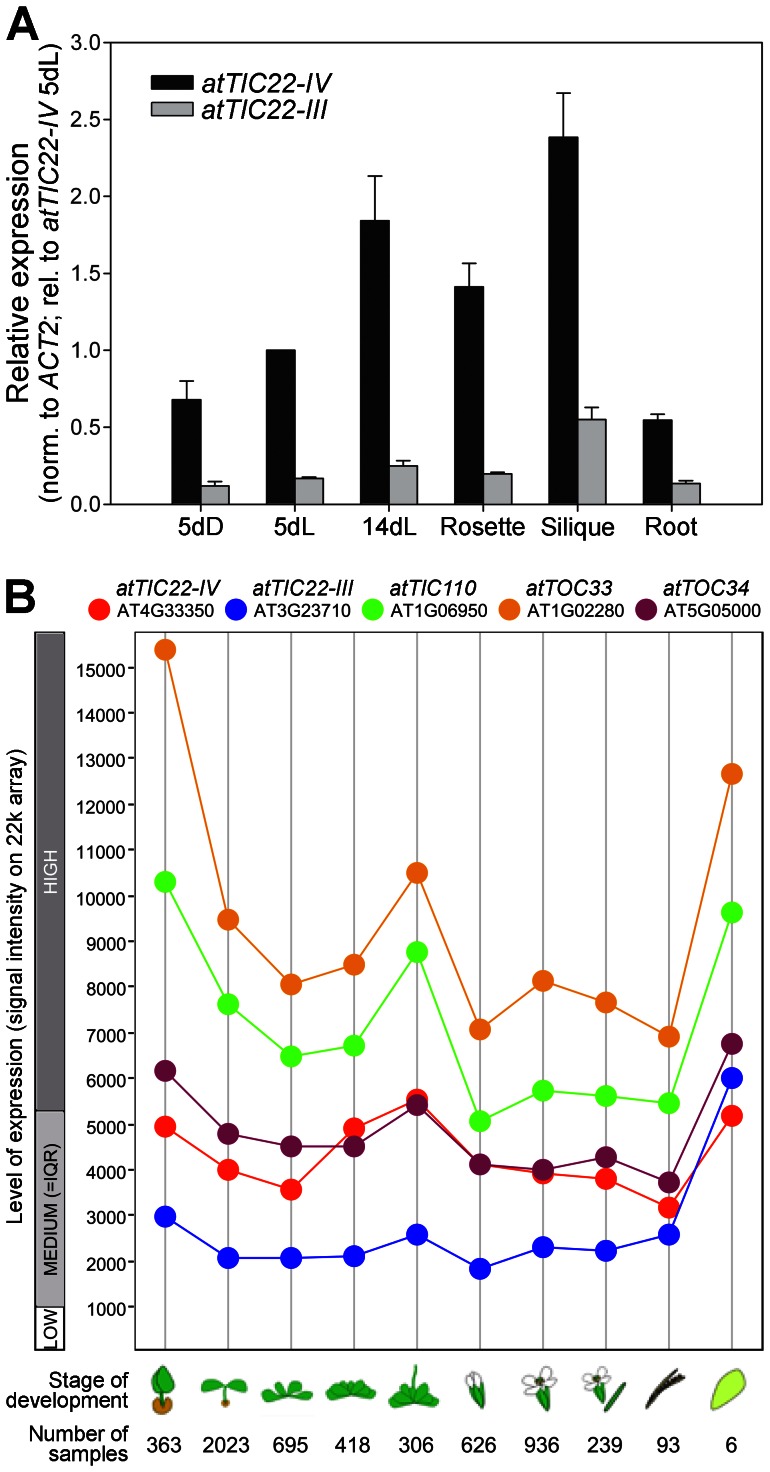
Expression of the Tic22 genes in different tissues and at different developmental stages. A. Quantitative RT-PCR analysis of total-RNA from whole seedlings grown for five days in the dark (5dD), or five and 14 days in the light (5dL and 14dL, respectively), as well as from three different tissues of mature plants (rosette leaves, siliques, and roots). RNA samples were representative of ∼10–30 seedlings (5dD, 5dL and 14dL), or 5–25 mature plants (rosettes, siliques and roots). Tic22 data were normalized relative to the control gene, *ACTIN2* (At3g18780), and then expressed relative to the *atTIC22-IV* 5dL value. Data shown are means (± SE) derived from three biological replicates. B. Affymetrix GeneChip data were analysed and retrieved using the Genevestigator V3 analysis tool (https://www.genevestigator.com) [29], [60]. Presented data were prepared using the Development representation in scatter-plot format. Data from all high-quality ATH1 (22 k) arrays were analysed; this amounted to a total of 7392 samples. Values shown are means. The total number of samples used to derive each data point shown is indicated. Typical ranges of low, medium, and high expression for the array type are shown; medium is defined as the interquartile range (IQR). Stages of development are defined as follows, from left to right: germinating seed, seedling, young rosette, developed rosette, bolting, young flower, developed flower, flowers and siliques, mature siliques, and senescence. Data representations were exported from Genevestigator in portable document format, and then annotated using appropriate graphics software. The genes analyzed were as follows: *atTIC22-IV* (At4g33350; red); *atTIC22-III* (At3g23710; blue); *atTIC110* (At1g06950; green); *atTOC33* (At1g02280; orange); *atTOC34* (At5g05000; purple).

To begin to elucidate the functions of the Arabidopsis Tic22 homologues, their developmental and tissue-specific gene expression patterns were studied by quantitative real-time RT-PCR (Figure 2A). The data indicated that *atTIC22-IV* is expressed, on average, at ∼5–6-fold higher levels than *atTIC22-III*, which is broadly consistent with the results of Rudolf et al. [17]. Indeed, database searches using the BLAST program [28] detected 20 expressed sequence tags (ESTs) for *atTIC22-IV* and only 7 for *atTIC22-III*. Qualitatively, however, the two genes displayed broadly similar expression profiles, with the highest levels occurring in samples expected to exhibit high levels of photosynthetic activity (i.e., 14-day-old light-grown seedlings, and rosette leaves).

To confirm the aforementioned observations, publicly-available microarray data were analysed using the Genevestigator tool [29], [30] (Figure 2B). This confirmed that *atTIC22-IV* is more highly expressed than *atTIC22-III*, and again indicated that highest levels of expression, for both genes, occur in photosynthetic tissues. Thus, the data imply that Tic22 is particularly important during photosynthetic development, when large numbers of proteins must be imported by developing and actively photosynthesizing chloroplasts. Further analyses of the expression data that are accessible using Genevestigator revealed that the Tic22 genes are not particularly responsive to biotic and abiotic stresses or other perturbations (data not shown).

### Identification and Analysis of Arabidopsis Tic22 T-DNA Insertion Mutants

**Figure 3:**
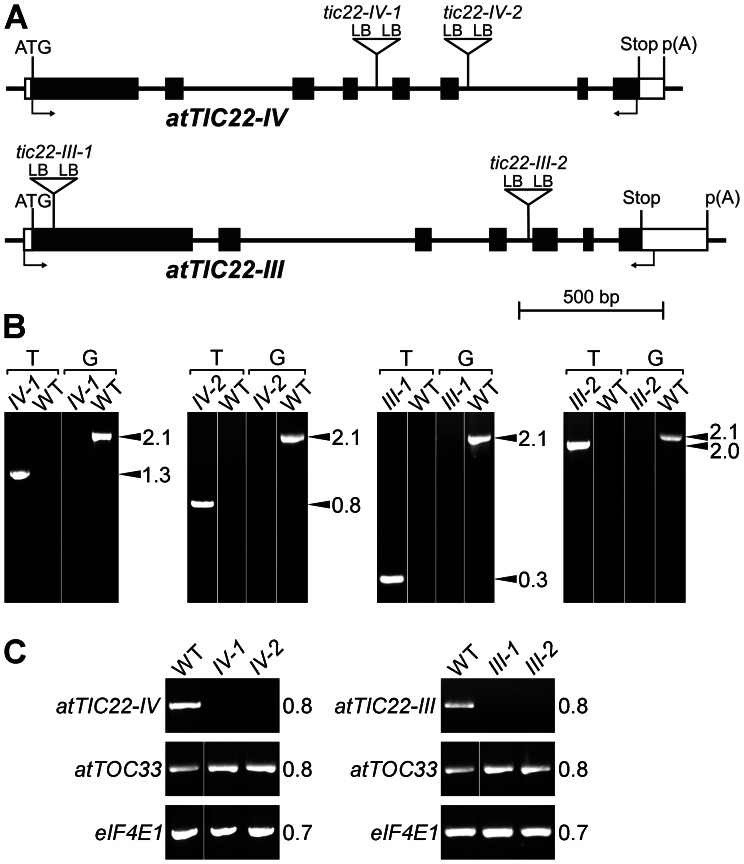
Molecular characterization of the Tic22 T-DNA insertion lines. A. Diagrams showing the structure of each gene and the locations of T-DNA insertions. Protein-coding exons are represented by black boxes, untranslated regions by white boxes, and introns by thin lines between the boxes. Locations of RT-PCR primers are shown by arrows beneath each gene model. T-DNA insertion sites are indicated precisely, but insertion sizes are not to scale. ATG, translation initiation codon; Stop, translation termination codon; p(A), polyadenylation site; LB, T-DNA left border. B. Genomic DNA samples extracted from wild type and putative homozygous mutants were analysed by PCR. Appropriate T-DNA- and *atTIC22*-gene-specific primers were employed. Two different primer combinations were used in each case: the first (“T”) comprised one T-DNA border primer and one gene-specific primer (LB+reverse: *tic22-1V-1*, *tic22-IV-2*, *tic22-III-2*; LB+forward, *tic22-III-1*); the second (“G”) comprised two gene-specific primers flanking the T-DNA insertion site. The PCR products were resolved by agarose gel electrophoresis, and visualized by staining with SYBR Safe. Amplification using “T” indicated the presence of the mutant allele, whereas amplification using “G” indicated the presence of the wild-type allele; amplification with the former but not the latter demonstrated that the plant was homozygous mutant. The genotype names are shortened as follows: “*22-IV-1″* indicates *tic22-IV-1*; “*22-IV-2″* indicates *tic22-IV-2*; and so on. Sizes of the amplicons are indicated at right (in kb). C. Expression of Tic22 genes in wild-type and mutant plants was analysed by RT-PCR. Locations of the amplification primers are shown in A. Similar analysis of *atTOC33* and of the translation initiation factor gene *eIF4E1* (At4g18040) was used to normalize loading. Amplicon sizes are indicated at right (in kb). RNA samples were from whole, 10-day-old homozygous plants grown *in vitro*, and were representative of ∼20–30 seedlings. PCR amplifications were performed over 25 cycles.

To elucidate the functional importance of the Arabidopsis Tic22 homologues *in vivo*, we identified two independent T-DNA insertion mutants for each of the two genes. All of the T-DNA insertion sites were confirmed by genomic PCR, and by the sequencing of the T-DNA/gene junctions at both sides in each case, as indicated (Figure 3A). Segregation analysis was performed to ensure identification of only single-locus insertion lines; Mendelian ratios of three antibiotic-resistant plants to one antibiotic-sensitive plant indicated the presence of single T-DNA insertions (Table S2). Further segregation analysis identified homozygous lines for analysis, and the zygosity of these was confirmed by genomic PCR (Figure 3B).

To assess the effect of each T-DNA insertion on *TIC22* gene expression, RT-PCR analysis was conducted in each case (Figure 3C). The results confirmed that the relevant full-length mRNA was absent for all of the mutants and so they were considered to be knockout alleles. Three of the mutants we selected for analysis on the basis of these results (*tic22-IV-2*, *tic22-III-1* and *tic22-III-2*) are equivalent to lines described by Rudolf et al. [17], who also concluded that these are null mutants.

### Phenotypic Analysis of the tic22 Single Mutants

**Figure 4:**
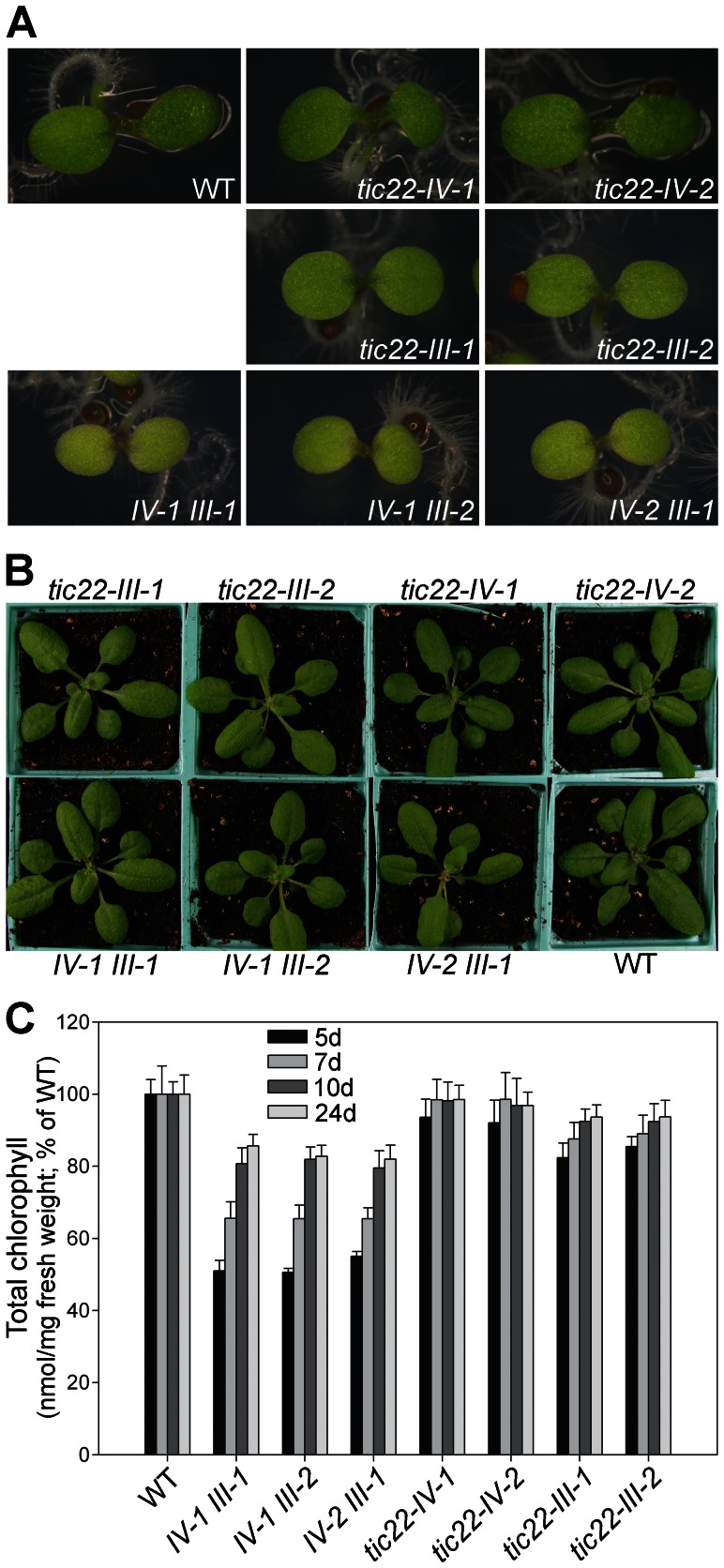
Phenotypic analysis of various *tic22* single- and double-mutant plants. A, B. Homozygous plants of the indicated genotypes were grown side-by-side, *in vitro*, for five days (A), or for 14 days prior to transferral to soil and growth for an additional 10 days (B). Representative plants are shown. C. Chlorophyll concentrations in 5, 7, 10 and 24-day-old plants of the indicated genotypes are shown. The plants were grown *in vitro* or on soil as described in A, under identical conditions. Values shown are means (±SE) derived from measurements of 12–24 different samples in each case. Units are nmol chlorophyll *a*+*b* per mg fresh weight, but the data have been normalized to the wild-type value at each developmental time-point.

**Figure 5:**
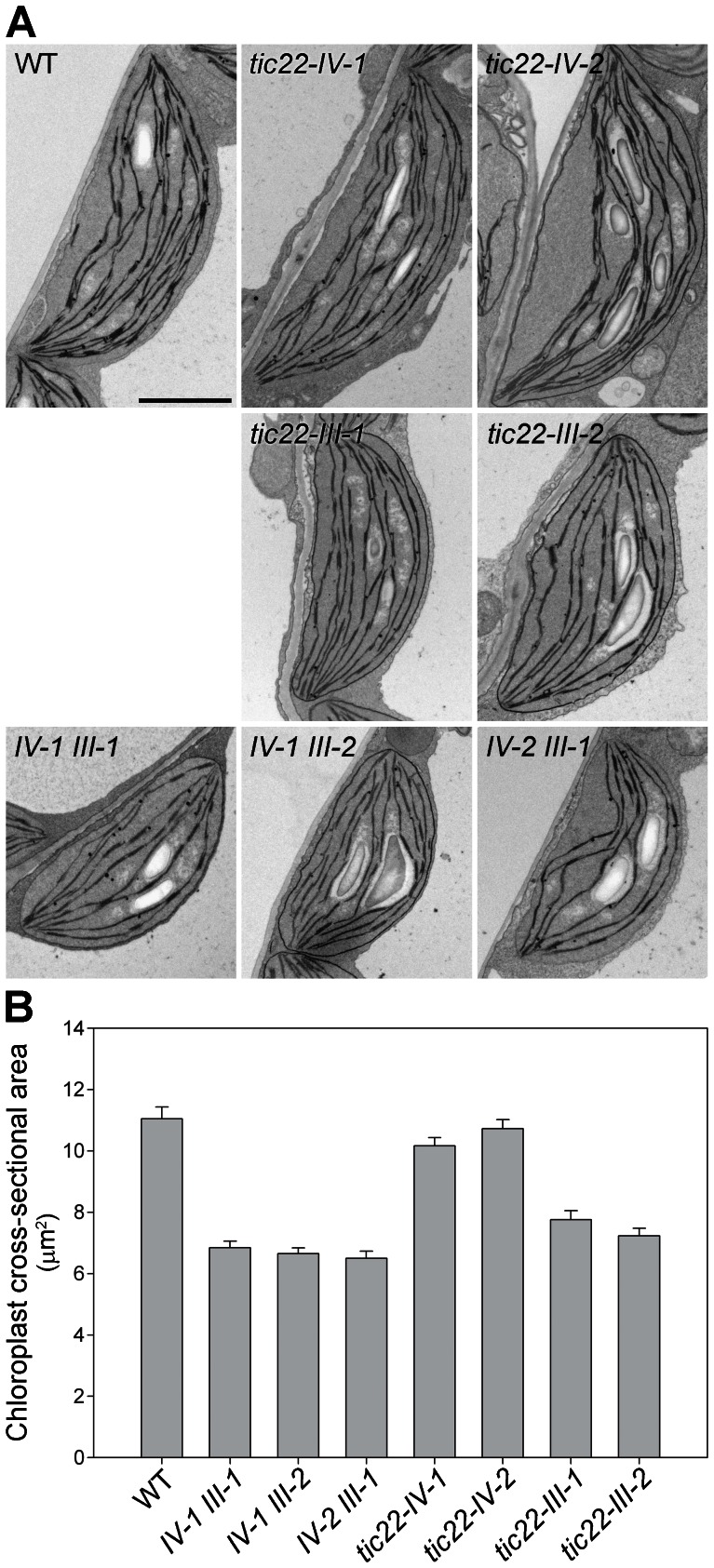
Analysis of plastid ultrastructure in the *tic22* single and double mutants. A. Cotyledons of 5-day-old plants were analysed by transmission electron microscopy. On average, ∼20 whole-chloroplast micrographs from each of three (two in the case of *tic22-IV-2 tic22-III-1*) independent plants per genotype (a minimum of 50 chloroplasts per genotype) were analysed for each genotype, and used to select the representative images shown. Size bar = 1.0 µm. B. Cross sectional area (µm^2^) of chloroplasts from the cotyledons of wild type and the *tic22* mutants was determined by the analysis of the micrographs described in A. At least 50 chloroplasts were measured for each genotype.

Typically, mutants lacking components of the chloroplast protein import machinery display obvious abnormal phenotypes, ranging from chlorosis to albinism or embryo lethality [24], [31], [32], [33]. However, none of the Tic22 single mutants displayed a particularly strong phenotype, and in fact the *tic22-IV* mutants were indistinguishable from wild type at all stages of development (Figure 4). That said, the *tic22-III* mutants did display a moderate but clear visible phenotype, and clear chlorophyll deficiency, particularly during the first week of development following germination (Figure 4A,C). This result was unexpected, as *atTIC22-III* is expressed at considerably lower levels than *atTIC22-IV* (Figure 2), while atTic22-III is less similar to the original Tic22 sequence from pea, as mentioned earlier. Moreover, Rudolf et al. [17] reported that all *tic22* single mutants were indistinguishable from wild type. In accordance with our observations, when the chloroplasts of the mutants were analysed by electron microscopy, those in *tic22-IV* were indistinguishable from wild-type organelles, whereas those in *tic22-III* were smaller with less well developed thylakoid networks (Figure 5).

### Analysis of *tic22* Double Mutant Plants

The phylogenetics analysis (Figure 1) suggested that the two Arabidopsis genes may be rather similar in function, implying redundancy between the homologues as an explanation for the lack of strong abnormal phenotypes in the single mutants. To test this possibility, the *tic22* single mutants were crossed in all pair-wise combinations, and the resultant F_2_ and F_3_ plants were analysed by scoring on antibiotic selective media, and by using diagnostic PCR tests similar to those shown in Figure 3B. In this way, we were able to identify three of the four possible double mutant combinations: *tic22-IV-1 tic22-III-1*, *tic22-IV-1 tic22-III-2* and *tic22-IV-2 tic22-III-1*. Unfortunately, the *tic22-IV-2 tic22-III-2* combination was not identified for technical reasons, but we concluded that three double mutants would be sufficient to enable reliable conclusions to be drawn, and so proceeded with the analysis.

As shown in Figure 4A, all three double mutants exhibited a very clear chlorotic phenotype during early development, and were additionally smaller in size than control plants. This phenotype was similar to that seen in the *tic22-III* single mutants, but considerably more severe. Chlorophyll deficiency in 5-day-old double-mutant plants was ∼50%, but in the *tic22-III* single mutants it was only 15–20% (Figure 4C). Chlorosis was not restricted to the cotyledons, which was evident upon inspecting the true leaves of 14-day-old plants (Figure S3). As the plants grew older, the paleness of the double mutants became less pronounced, and chlorophyll levels approached those in the wild type (Figure 4B,C). Electron microscopy data were consistent with the chlorophyll data and visible phenotypes, revealing that chloroplasts in young double-mutant plants are considerably smaller and less developed internally than those in wild type or even the *tic22-III* single mutants (Figure 5).

These results are therefore consistent with the notion that the two Arabidopsis proteins share considerable functional redundancy.

### Assessment of Plastid Protein Levels in the *tic22* Double Mutants

**Figure 6:**
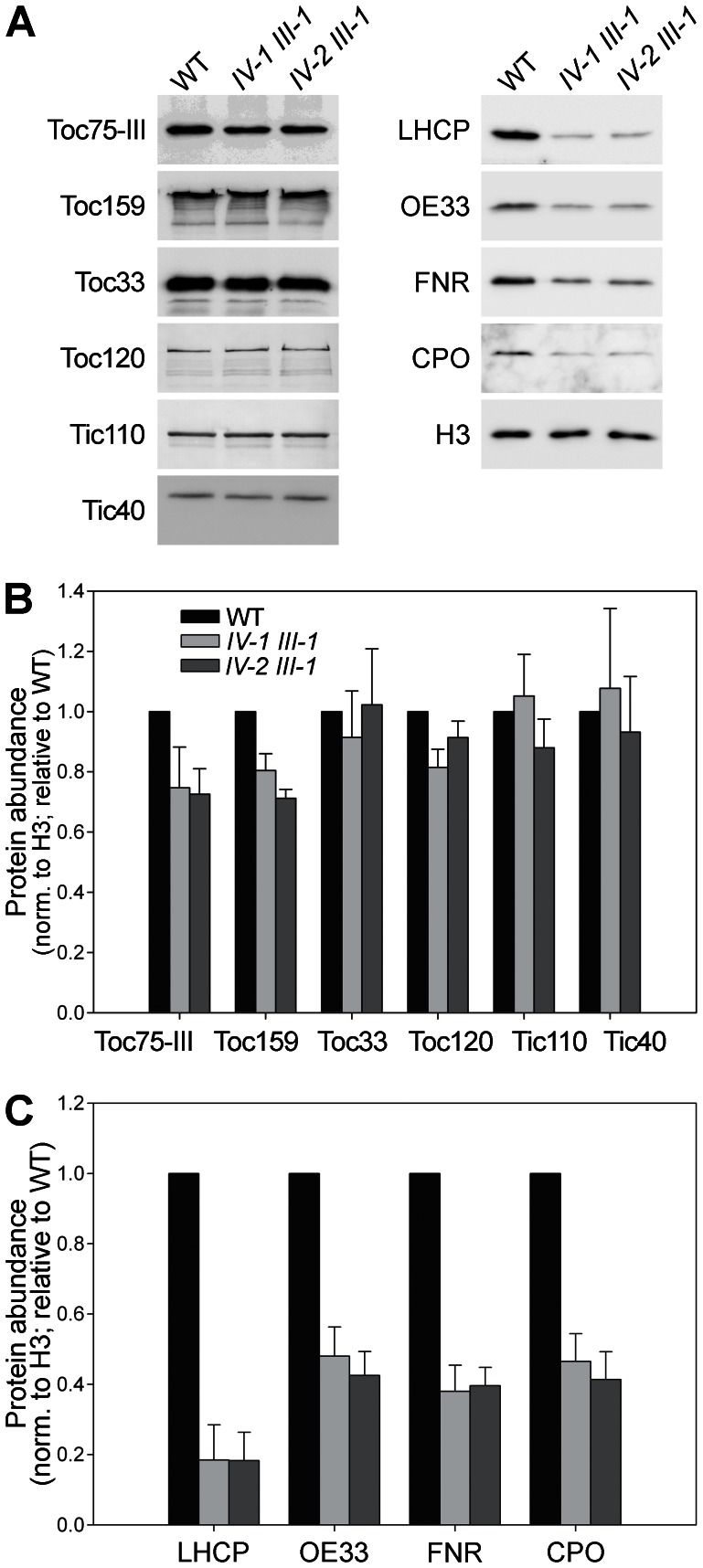
Chloroplast protein levels in the *tic22* double mutants. A. Equal protein samples (20 µg) from 5-day-old homozygous, MS-grown plants of the indicated genotypes were analysed by immunoblotting using the antibodies shown. LHCP, light-harvesting chlorophyll-binding protein; OE33, oxygen-evolving complex 33 kD subunit; FNR, ferredoxin-NADP reductase; CPO, coproporphyrinogen oxidase; H3, histone H3. B,C. Chloroplast protein bands in A (as well as those from other, similar experiments) were quantified using Aida software (Raytest), and then the data were normalized using equivalent H3 data. Values shown are means (±SE) derived from 3–8 independent measurements.

To further characterize the chloroplast biogenesis defects in the *tic22* double mutants, the levels of several chloroplast proteins were analysed by immunoblotting in young seedlings. As the three double mutants appeared to be phenotypically identical (Figures 4 and 5), we focused this analysis on just two of them. We analysed proteins of the TOC/TIC import machinery, as well as various components of the photosynthetic or biochemical apparatus (Figure 6).

As was observed previously in mutants lacking another TIC component (Tic20) [33], levels of most of the translocon components investigated were not obviously affected. That said, the abundance of the outer membrane channel protein atToc75-III (as well as one of its major partners, the receptor protein atToc159) was slightly reduced in the mutants. The effect on atToc75-III might indicate a role for Tic22 in the biogenesis of this (and perhaps other) beta-barrel proteins, as has been reported for a cyanobacterial Tic22 homologue [13]; alternatively, it may simply be an indirect effect linked to the general disruption of chloroplast development. Lack of an effect on the TIC proteins, atTic110 and atTic40, was somewhat surprising, as their precursors pass through the TIC machinery en route to the inner membrane [34], [35]. As was discussed previously [33], it is possible that compensatory mechanisms, such as reduced protein turnover, are implemented in the mutants to help maintain the levels of these TIC proteins.

Much stronger effects of the *tic22* mutations were observed for components of the photosynthetic apparatus (LHCP, OE33 and FNR), with the reductions relative to wild type ranging from 50% to 80%. The tetrapyrrole biosynthetic enzyme, CPO, was also strongly depleted (by ∼50%) in the double mutants. Together, these results provide further evidence that chloroplast biogenesis is substantially disrupted by the loss of Tic22, supporting the notion that this component is important for chloroplast protein import.

### Analysis of Chloroplast Protein Import in the *tic22* Double Mutants

**Figure 7:**
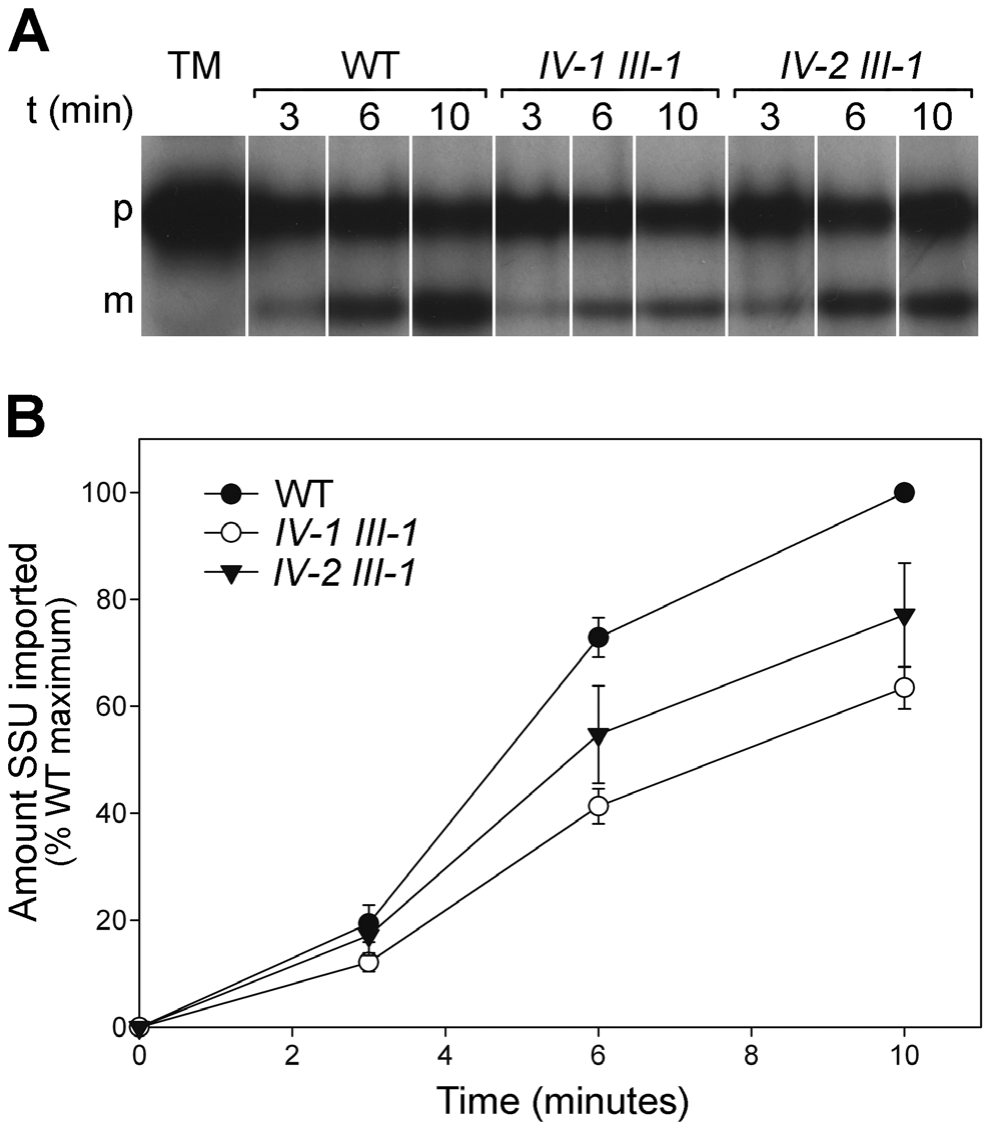
Analysis of chloroplast protein import efficiency in the *tic22* double mutants. A. Chloroplasts were isolated from 14-day-old, *in vitro*-grown plants of the indicated genotypes, and used in protein import assays with [^35^S]-methionine-labelled Rubisco SSU preprotein. Import was allowed to proceed for 3, 6 and 10 minutes, as indicated, and then samples were analysed by SDS-PAGE and fluorography. TM indicates an aliquot of the SSU translation mixture equivalent to 10% of the amount added to each assay; p and m indicate the precursor and mature forms of SSU, respectively. B. Mature protein bands observed in A were quantified using ImageQuant software, and then the data were expressed as percentages of the value for the final, wild-type time-point. These data, together with those from two additional, similar experiments, were used to calculate the mean (±SD) values shown (n = 3).

The experiments described so far imply that Tic22 plays a significant role in preprotein import. To obtain more direct evidence in support of this notion, we analysed chloroplast protein import efficiencies in two of the double mutants, using isolated chloroplasts and the precursor of the Rubisco small subunit (SSU), which is a widely-used model preprotein. Time-course import experiments were carried out, and then import was quantified by measuring the amount of mature protein in the chloroplasts at each time-point. As shown in Figure 7, import of SSU was reduced in both of the *tic22* double mutants analysed: on average, the maximal amount of imported protein was down to ∼70% of the wild-type level in the mutants. Analysis of the data from repeated experiments (at the 6 and 10 minute time-points) using a Student’s t-test showed that the reduction in import, relative to wild type, was significant for both of the mutants (p<0.05). Moreover, even though slightly more imported protein was observed in the case of *tic22-IV-2 tic22-III-1*, the datasets for the two double mutants were not significantly different from each other (p>0.05). Thus, these results provide strong support for the hypothesis that Tic22 plays an important role in plastid preprotein import.

## Discussion

Our phylogenetic analysis provided strong support for a cyanobacterial origin of the Tic22 gene family. A functional relationship between atTic22-IV and a homologous protein in the cyanobacterium *Anabaena sp.* PCC 7120 (sequence named Alr0114) was also shown by Tripp et al. [13], which further supports this conclusion. Moreover, the fact that Tic22-related sequences are found in all investigated organism groups having primary chloroplasts, as well as in groups with chloroplasts derived from secondary endosymbioses [15], implies that Tic22 constitutes an important part of the protein translocon complex of chloroplasts.

Phylogenetic analysis of a large number of sequences revealed that a duplication happened in the Tic22 gene family at about the time when land plants first emerged. This event resulted in two evolutionarily conserved groups of proteins (characterized by atTic22-III and atTic22-IV) (Figure 1). Estimates of branch lengths did not reveal any significant differences between the two clades indicative of shifts in evolutionary rates, suggesting that a functional differentiation between the two groups has not occurred. Indeed, analyses of single- and double-mutant Arabidopsis plants lacking these proteins were consistent with the notion that they are functionally redundant.

Our analysis also infers that a partial gene duplication happened in the gene family after the split between red algae and other archaeplastida. This event resulted in a clade of proteins with unknown function (including the Arabidopsis sequence At5g62650) that has been conserved in all investigated land plant species. No putative orthologues were found in the investigated algal species, although the phylogenetic position of this clade indicates that orthologues could be present in green algae. Further analyses are required to gain better understanding of when this gene duplication happened, and this will be greatly facilitated by the release of more whole genome sequence data from the relevant algal groups.

We showed that the two Arabidopsis Tic22 genes are expressed throughout development, and with similar expression profiles. However, expression levels of *atTIC22-IV* were generally much higher than those of *atTIC22-III* (Figure 2). When considered in conjunction with our phenotypic analysis of the *tic22* single mutants (Figure 4), this is a curious result, as it is the *tic22-III* mutants that display significant developmental defects (the *tic22-IV* mutants are indistinguishable from wild type throughout development). Our phylogenetic and genetic analyses suggested that the two homologues may be functionally similar; however, the apparent discrepancy between expression levels and mutant phenotype severity implies that the two proteins have in fact diverged functionally to some extent, with the result that the atTic22-III isoform is now a more active or efficient protein, or has differing client specificity. Explanations that cannot be eliminated at this stage include possible differences in translation efficiency or protein stability.

Loss of either Tic22 isoform, individually, does not have a major impact on chloroplast development in Arabidopsis. However, when both genes are knocked out, an important role in chloroplast development is clearly revealed, particularly in very young plants (Figures 4 and 5). However, as the plants grow older, the severity of the double mutant phenotypes decreases markedly, and can be quite difficult to detect in mature plants. This does not seem to reflect expression differences, as neither of the genes is expressed at much higher levels during early development compared with later stages. A plausible explanation is that the role of Tic22 is only critical during early stages of chloroplast biogenesis, when protein import rates are especially high as the photosynthetic apparatus is being established [36]. Declining phenotype severity might also reflect the possibility that Tic22’s role in import is to increase the efficiency of a process that can nonetheless proceed in its absence. Thus, chloroplast biogenesis might proceed without Tic22, but at a slower rate, so that eventually such plants are able to “catch-up” with the wild type.

One possible role for Tic22 that might conform to the above criteria is the chaperone function that was proposed in relation to the cyanobacterial and apicomplexan homologues [13], [15]. In this regard, it is interesting to note that levels of the Omp85-related protein, Toc75, are noticeably reduced in the *tic22* double mutants. This is consistent with the notion that Tic22 plays a chaperone-like role in the passage of proteins such as Toc75 through the inter-membrane space [13]. However, it seems unlikely that the phenotype of the *tic22* double mutants can be attributed entirely to Toc75 deficiency, as the *toc75-III-3* mutant (which is considerably more Toc75-deficient [37]) is visibly greener than the *tic22* mutants during early development [38], [39]. Moreover, chloroplast protein levels are more severely reduced in the *tic22* mutants (Figure 6, this study) than in the *toc75-III-3* mutant (Q. Ling, unpublished observations). Thus, the data are consistent with a more general chaperoning role for plant Tic22 in the transport of a range of different preproteins.

Previous work showed that the chloroplast import of Tic22 depends on its N-terminal presequence [9]. However, processing of preTic22 was seen to be a slow event in the import process, with large amounts of imported preTic22 remaining uncleaved in envelope [9]. It was proposed that Tic22 is processed in the intermembrane space by an unknown peptidase, and that its targeting pathway is different from other known routes [9], [10]. These observations may account for our failure to observe efficient chloroplast targeting of a range of different full-length and truncated Tic22 fusions to YFP in transfected protoplasts. It is conceivable that this unusual import pathway is unable to accommodate such heterologous passenger proteins, or that the C-terminal addition of a large tag like YFP somehow disrupts the targeting signals of the Tic22 protein. Regardless, there is little doubt that the Arabidopsis Tic22 proteins are chloroplast localized, as this has been shown by *in vitro* import (this study), protease treatment of isolated chloroplasts [17], and by the proteomic analysis of purified chloroplast fractions [25], [26], [27].

The data presented in this report complement those of Rudolf et al. [17] in the following ways: we conducted a comprehensive analysis of the evolution of the Tic22 gene family; we identified and characterized a *tic22-III* single mutant phenotype; we identified and characterized more than one *tic22* double-mutant genotype; we analysed the accumulation of a range of different chloroplast proteins in the *tic22* mutants by immunoblotting; we conducted a quantitative analysis of chloroplasts using electron microscopy in single- as well as double-mutant plants. In addition, our results provide a robust confirmation of many of those reported by Rudolf et al. [17].

## Materials and Methods

### Phylogenetic Analysis

The whole genome sequences (WGS) used in this investigation are listed in Table S1, and the python code used to automate the various analysis steps is available at https://github.com/mtop. Detailed descriptions of each analysis step are available at www.matstopel.se/notebook and the resulting files can be found at https://github.com/mtop-data/tic22.

We downloaded the amino acid sequences of the 31 plant WGS datasets of Phytozome v.8.0, as well as of the early-release genome of *Panicum virgatum*, from www.phytozome.net [40]. In addition, WGS datasets from *Cyanidioschyzon merolae* (http://merolae.biol.s.u-tokyo.ac.jp/) and *Cyanophora paradoxa* (http://cyanophora.rutgers.edu/cyanophora/home.php), and a partial EST library of *Marchantia polymorpha* (http://www.genome.jp/dbget-bin/www_bget?gn:T20061), were also downloaded. Each of the 35 plant genomes (including the *A. thaliana* dataset) was then searched in separate BLAST+ v.2.2.26 [41] analyses, using atTic22-III, atTic22-IV and At5g62650 (NCBI accession numbers NP_189013.1, NP_195061.1 and NP_568958.1, respectively) as query sequences. The five best matches were saved and aligned to the respective query sequences. This process was automated using the python script “blast_and_align.py”. Each alignment result was then inspected using SeaView v.4.3.5 [42], to identify putative homologous sequences for further analysis. It is worth noting that, when using atTic22-III and atTic22-IV as query sequences, this method did not identify At5g62650, a third Arabidopsis sequence that we believe originates from a partial gene duplication.

To identify an outgroup dataset, we performed a reciprocal BLAST analysis (using the python script “reciprocal_blast.py”) of 67 cyanobacterial WGS datasets, and 54 WGS datasets from other groups of bacteria, using atTic22-III and atTic22-IV as query sequences. This analysis identified 32 cyanobacterial sequences and three sequences from other bacterial groups with a reciprocal best match to either of the query sequences. Further “blast_and_align.py” analyses of the genome sequences from bacterial groups outside cyanobacteria were then performed. This analysis found no convincing evidence suggesting an origin of the gene family in any bacterial group other than cyanobacteria.

Sequences from the 20 plant and algal species and five cyanobacterial species listed in Table S1 were then selected for the final phylogenetic analysis. Considering the available WGS datasets, we believe that these species are a representative set for the viridiplantae tree of life. The 58 amino acid sequences selected (Table S1) were then aligned using mafft-linsi v6.864b [43]. The resulting alignment was analysed using Zorro [44] to evaluate the quality of the alignment. Columns with a probability score of 0.4 or higher were then analysed using MrBayes v.3.2 [45] and a mixed amino acid model. The tree was rooted using the *Gloeobacter violaceus* sequence as outgroup, and two independent analyses were run simultaneously for 20,000,000 generations (average standard deviation of split frequencies was 0.006 at the end of the analysis) with four chains each, and trees were sampled every 1,000 generations. Convergence of the analysis was examined using Tracer v.1.5 [46], and a 50% majority-rule consensus phylogram was produced using the default settings.

### Plant Growth Conditions

All *Arabidopsis thaliana* plants were Columbia-0 (Col-0) ecotype, and were grown as described previously [47]. To select for T-DNA insertions, antibiotics were added to the Murashige-Skoog (MS) medium: kanamycin monosulfate, 50 µg/ml (*tic22-IV-1*); sulfadiazine, 11.25 µg/ml (*tic22-IV-2*, *tic22-III-1* and *tic22-III-2*). For the expression analysis (Figure 2A), wild-type Col-0 was grown on MS medium (all seedling samples), or was sown and grown on soil for the harvesting of rosette leaves and siliques from 28-day-old plants; roots were harvested from 28-day-old MS-grown plants. All plants were grown under a long-day cycle (16 hours light, 8 hours dark).

### Subcellular Localization Analysis by Protoplast Transfection

Full-length atTic22 coding sequences lacking the native stop codon were amplified using primers that introduce partial attB recombination sites. The primers were as follows (the 5′ primer appears first in each case; the first and last native codons are indicated in bold text): *atTIC22-IV*, 5′-AA AAA GCA GGC TCC **ATG** GAG TCA TCA GTG AAA C-3′ and 5′-A GAA AGC TGG GTT **CTC** TTT GAT CAA ATC CTG-3′; *atTIC22-III*, 5′-AA AAA GCA GGC TCC **ATG** AAT TCA AAC ATT TTC CCA CC-3′and 5′-A GAA AGC TGG GTT **CTC** CTG TGT TTG CTC AGT TG-3′.

In addition to the full-length clones, we generated two C-terminal truncations for each gene. The forward primers for each one were the same as used for the full-length constructs above. The reverse primers were as follows: *atTIC22-IV-S*, 5′-A GAA AGC TGG GTT **AGC** TTT AGC GAC AAG AGA TGG-3′; *atTIC22-IV-L*, 5′-A GAA AGC TGG GTT **TTG** TTG ATC TCC TCT TGA TGC-3′; *atTIC22-III-S*, 5′-A GAA AGC TGG GTT **TCT** CTC CTC AAT AGC ATC AGC-3′; *atTIC22-III-L*, 5′-A GAA AGC TGG GTT **ATC** GCC TGG TTT CAG AGC AGG-3′.

Secondary amplification using the following primers (for both genes) completed the terminal attB sites: 5′-GGG GAC AAG TTT GTA CAA AAA AGC AGG CT-3′ and 5′-GGG GAC CAC TTT GTA CAA GAA AGC TGG GT-3′.

The amplified sequences were then inserted into the p2GWY7 vector [23] to make C-terminal YFP fusions by Gateway recombination cloning (Invitrogen, Paisley, UK), via the pDONR201 donor vector. Protoplasts were prepared from 14-day-old, wild-type Arabidopsis plants, and transfected, as described previously [48], [49]. Fluorescence microscopy employed a Nikon Eclipse TE-2000E inverted fluorescence microscope equipped with filters for analysing YFP (exciter HQ500/20x, emitter HQ535/30m) and chlorophyll autofluorescence (exciter D480/30x, emitter D660/50m) (Chroma Technologies, Rockingham, VT, USA).

### Quantitative Real-time PCR (QPCR) and Standard RT-PCR

Total-RNA was isolated using an RNeasy Plant Mini Kit (Qiagen, Hamburg, Germany), and treated with DNAse I (DNA-free; Ambion, Austin, TX, USA). Reverse transcription was performed as described previously [50], [51]. For QPCR, three biological replicates were analysed, and each one was measured in triplicate using an MJ Research Chromo4 Gradient Cycler (Bio-Rad, Hercules, CA, USA) and SYBR Green Jump Start Taq Ready Mix (Sigma, St Louis, MO, USA). Data were normalized using similarly-derived *ACTIN2* (At3g18780) data. Average values for each biological replicate were calculated, and then these were used to derive the presented means. The QPCR primers used were: *atTIC22-IV* sense, 5′-GAG TCA TCA GTG AAA CCC AAT C-3′; *atTIC22-IV* antisense, 5′-GGC GGA GTA GGA AGA GAG AAA C-3′; *atTIC22-III* sense, 5′-GTC TCA AGC ATC ATT TTC CCG AG-3′; *atTIC22-III* antisense, 5′-CGT GTT GGC GTT GAA GAG AGG-3′; *ACTIN2* sense, 5′-TCA GAT GCC CAG AAG TGT TGT TCC-3′; *ACTIN2* antisense, 5′-CCG TAC AGA TCC TTC CTG ATA TCC-3′.

For standard RT-PCR (Figure 3C), total-RNA isolation (from ∼20–30 10-day-old, homozygous seedlings grown *in vitro*) and RT-PCR were performed as described above and previously [50], [51]. Control primers (*eIF4E1* and *atTOC33*) have been described previously [50]. The Tic22 primers used were: *atTIC22-IV* sense, same as for QPCR; *atTIC22-IV* antisense, 5′-CCT GCA TGT GTT GTG CAT AAC TTC-3′; *atTIC22-III* sense, same as for QPCR; *atTIC22-III* antisense, 5′-GAG GTT TTA CGA TGC TCC AAG G-3′. Products were resolved by electrophoresis and stained with SYBR Safe (Invitrogen). To avoid saturation, only 25 amplification cycles were employed; this was sufficient to produce a faint band after staining.

### Identification and Analysis of the Tic22 Mutants

The T-DNA insertion lines were obtained from the following sources. The *tic22-IV-1* was from the Salk Institute Genomic Analysis Laboratory (SIGnAL) via the Nottingham Arabidopsis Stock Centre (NASC) (line SALK_022794) [52]. The *tic22-IV-2*, *tic22-III-1* and *tic22-III-2* mutants were from Genomanalyse im Biologischen System Pflanze-Kölner Arabidopsis T-DNA (GABI-Kat; lines 710-E01, 387-C03 and 518-B03, respectively) [53]. The GABI-Kat lines were also described by Rudolf et al. [17]: the *tic22-IV-2*, *tic22-III-1* and *tic22-III-2* mutants described here are respectively equivalent to the *tic22-IV-1*, *tic22-III-2* and *tic22-III-1* mutants reported previously [17].

Mutant genotypes were assessed by PCR (Figure 3B). Genomic DNA was extracted from plants [54] and then analysed by PCR using standard procedures. The gene-specific primers used were the sense and antisense primers used for the standard RT-PCR analysis. The T-DNA-specific primers used were: *tic22-IV-1* left border (LB), 5′-GCG TGG ACC GCT TGC TGC AAC T-3′; *tic22-IV-2*, *tic22-III-1* and *tic22-III-2* LB, 5′-CCC ATT TGG ACG TGA ATG TAG ACA C-3′.

Amplification products were resolved by agarose gel electrophoresis and stained with SYBR Safe. The location of each T-DNA insertion was determined precisely (Figure 3A) by the sequencing of junction-spanning PCR products.

### Electron Microscopy

Transmission electron microscopy was performed as described previously [55] with the following minor modification: en-bloc staining with uranyl acetate was omitted. Mid-lamina cross-sections of the cotyledons of plants grown *in vitro* for five days were analysed. The length and width of each chloroplast was measured using Adobe Photoshop software, using the measure tool; these values were then used to estimate organelle cross-sectional area using the following formula: π × 0.25 × length × width. In all cases, reference to an internal standard was used to convert the values into suitable units of length or area. Procedures were carried out at the Electron Microscopy Laboratory, Faculty of Medicine and Biological Sciences, University of Leicester.

### Chloroplast Protein Import Assays

Chloroplasts from Arabidopsis were isolated and the import reactions were carried out according to Aronsson and Jarvis [47], [56]. Briefly, each import reaction contained 10 million chloroplasts, 5 mM MgATP, 10 mM methionine, and translation mixture not exceeding 10% of the total volume, and was carried out at 25°C in white light (100 µmol/m^2^/s) for different time periods. Samples were resolved on SDS-PAGE gels [57], fixed, and exposed to Hyperfilm MP (GE Healthcare, Chalfont St Giles, UK). Quantification of the import assays was performed using ImageQuant software (GE Healthcare).

*In vitro* transcription/translation was performed using a coupled TNT system (Promega, Madison, WI, USA) based on rabbit reticulocyte lysate containing [^35^S]-methionine and T7 RNA polymerase, according to the manufacturer’s instructions (Promega). Using M13 primers, the template DNA for the transcription/translation reactions was amplified by PCR from Arabidopsis cDNA clones for the precursors of Rubisco small subunit 1A and atTic22-III, according to Aronsson and Jarvis [56].

Chloroplast incubations with thermolysin contained 50 µg/ml thermolysin and 300 µM CaCl_2_, and reactions were conducted for 5 min on ice [24]. Control reactions had the thermolysin replaced with an equal volume of import buffer, and were similarly incubated. All reactions were terminated by addition of an equal volume of 50 µM EDTA. Trypsin reactions contained 50 µg/ml trypsin, and were conducted for 30 min on ice [58]. Control trypsin reactions had the trypsin solution replaced with an equal volume of import buffer, and were similarly incubated. The reactions were stopped by adding trypsin inhibitor to a final concentration of 10 µg/ml.

### Immunoblotting

Immunoblotting employed previously described procedures [50], [51], with minor modifications. Leaf material (∼100 mg) from 5-day-old plants grown on MS plates was ground in liquid nitrogen and extracted with 200 µL buffer (100 mM Tris-HCl, pH 6.8, 10% [v/v] glycerol, 0.5% [w/v] SDS, 0.1% [v/v] Triton X-100, 10 mM DTT, 5 mM EDTA; 10 µL protease inhibitor cocktail [Sigma] was added per 1 mL buffer). Insoluble material was removed by centrifugation at 20,000 *g* for 10 min at 4°C, and 150 µL of the supernatant (extract) was retained for analysis. All protein extracts were quantified against BSA standards using Bradford reagent (Bio-Rad) prior to analysis. Samples (20 µg per lane) were diluted two-fold in 2× SDS-PAGE sample buffer, and separated by SDS-PAGE [57]. Separated proteins were stained with Coomassie Brilliant Blue R250 (Fisher Scientific, Loughborough, UK), or blotted onto Hybond ECL membrane (GE Healthcare).

Primary antibodies were polyclonal antisera raised in rabbits against: atToc75-III POTRA-domain (residues 75–158); atToc159 A-domain [31]; atToc33 G-domain (residues 1–262); atToc120 A-domain (residues 1–343); atTic110 stromal domain [59]; atTic40 stromal domain (residues 130–447); LHCP (from pea); OE33 (from pea); FNR (from barley); CPO (from tobacco); and histone H3 (Abcam, Cambridge, UK). Antibodies not made in-house or purchased commercially were kindly provided by Bernhard Grimm (CPO), Neil Hoffman (LHCP, OE33), Felix Kessler (atToc159), and Henrik Scheller (FNR). Secondary antibodies were anti-rabbit IgG conjugated with alkaline phosphatase (Sigma), or with horseradish peroxidase (Santa Cruz Biotechnology, Heidelberg, Germany). In the case of the former, the detection reagent was BCIP/NBT alkaline phosphatase substrate (Sigma); in the case of the latter, the detection reagent was ECL Plus (GE Healthcare). Chemiluminescence detection employed a Fujifilm LAS-4000 imager. Quantification of all images was performed using Aida software (Raytest, Straubenhardt, Germany).

## Supporting Information

Figure S1 **Annotated alignment of the Arabidopsis and pea Tic22 proteins.** Full-length amino acid sequences of psTic22, atTic22-IV and atTic22-III, and exons 2–5 of At5g62650, were aligned by mafft [43]. Residues identical in sequences are highlighted in black, whereas similar residues are highlighted in grey. The locations of (predicted) transit peptide cleavage sites are indicated (the first mature residue is coloured green); the experimentally-determined cleavage site is shown for psTic22 [7], whereas TargetP-predicted sites are shown for the Arabidopsis proteins.
(TIF)

Figure S2 **Analysis of the localization of the Arabidopsis Tic22 proteins.** A, Analysis of Tic22:YFP fusions in transfected Arabidopsis protoplasts. Wild-type Arabidopsis protoplasts were transfected with the indicated plasmids (atTic22-IV[full-length]:YFP, atTic22-IV[1–224]:YFP and atTic22-IV[1–78]:YFP) and then analysed for YFP fluorescence (green, left panels) and chlorophyll autofluorescence (red, centre-left panels), as well as under brightfield illumination (right panels). An overlay of the YFP and chlorophyll images is presented (centre-right panels). Similar analyses of an equivalent series of three atTic22-III YFP constructs produced identical results (data not shown). Scale bar = 10 µm. B, Analysis of the localization of the Arabidopsis atTic22-III protein following *in vitro* import. Chloroplasts isolated from 14-day-old wild-type plants were used in protein import assays with either [^35^S]-methionine-labelled atTic22-III or similarly-labelled Rubisco small subunit (SSU) precursor as a control. All import reactions were allowed to proceed for 20 minutes. At the end of the import reactions, the chloroplasts were recovered and treated in the absence (−) or presence (+) of either thermolysin or trypsin, prior to analysis by SDS-PAGE and fluorography. TM indicates an aliquot of the atTic22-III or SSU translation mixture equivalent to 10% of the amount added to each assay; p and m indicate the precursor and mature forms of SSU, respectively. The mature from of Rubisco SSU is located in the stroma, and so is expected to be resistant to both proteases; on the other hand, un-imported SSU precursor is expected to be sensitive to both proteases. Concerning atTic22-III, resistance to thermolysin and sensitivity to trypsin is consistent with localization to the intermembrane space.
(TIF)

Figure S3 **Visible appearance of 14-day-old** ***tic22*** **double-mutant plants.** Homozygous plants of the indicated *tic22* double-mutant genotypes were grown alongside wild type *in vitro* for 14 days. Representative plants were then photographed. The images illustrate clearly that the chlorosis associated with loss of Tic22 is not restricted to the cotyledons, and can also be seen in true leaves.
(TIF)

Table S1 **The Tic22-related amino acid sequences used for the phylogenetic analysis.**
(XLS)

Table S2 **Segregation analysis of the Tic22 T-DNA mutant lines employed in this study.**
(XLS)
