# Supplementary figures and images for: Evolutionary, Molecular and Genetic Analyses of Tic22 Homologues in Arabidopsis thaliana Chloroplasts
Source: PLoS One. 2013 May 13;8(5):e63863. doi: 10.1371/journal.pone.0063863 (PMC3652856; doi:10.1371/journal.pone.0063863)

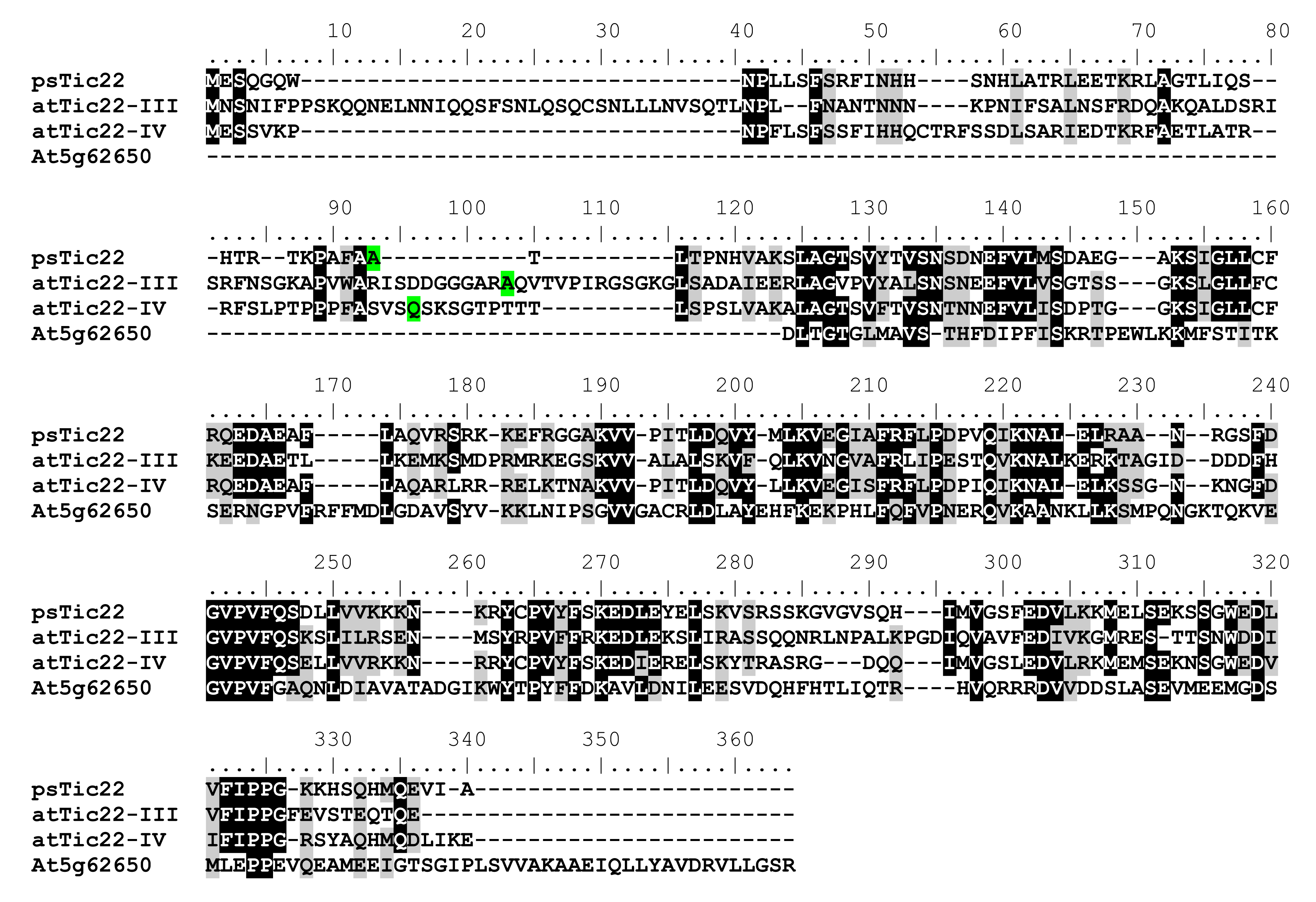

Supplement: Figure S1 — Annotated alignment of the Arabidopsis and pea Tic22 proteins. Full-length amino acid sequences of psTic22, atTic22-IV and atTic22-III, and exons 2–5 of At5g62650, were aligned by mafft [43]. Residues identical in sequences are highlighted in black, whereas similar residues are highlighted in grey. The locations of (predicted) transit peptide cleavage sites are indicated (the first mature residue is coloured green); the experimentally-determined cleavage site is shown for psTic22 [7], whereas TargetP-predicted sites are shown for the Arabidopsis proteins. (TIF) [file pone.0063863.s001.tif]

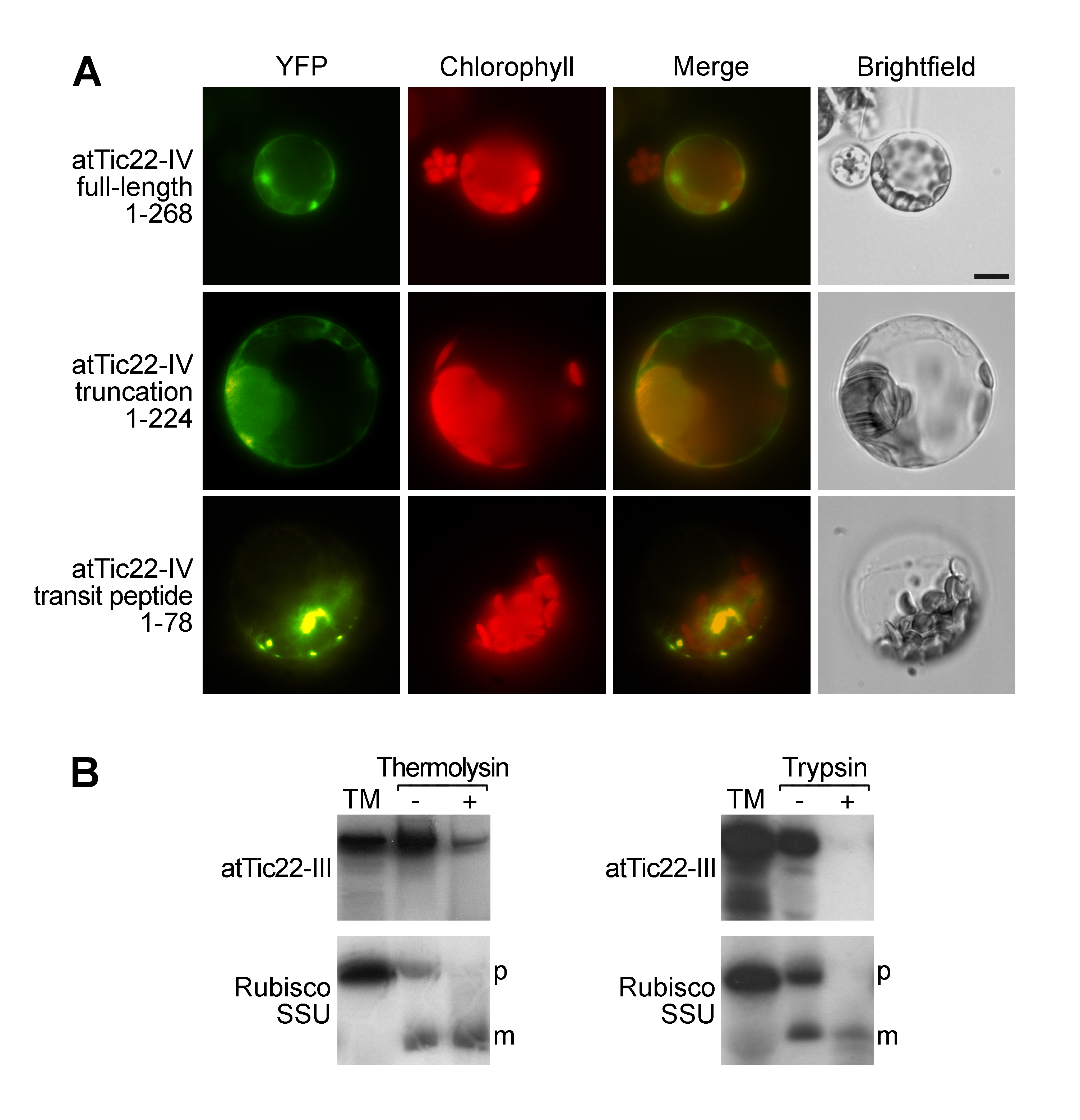

Supplement: Figure S2 — Analysis of the localization of the Arabidopsis Tic22 proteins. A, Analysis of Tic22:YFP fusions in transfected Arabidopsis protoplasts. Wild-type Arabidopsis protoplasts were transfected with the indicated plasmids (atTic22-IV[full-length]:YFP, atTic22-IV[1–224]:YFP and atTic22-IV[1–78]:YFP) and then analysed for YFP fluorescence (green, left panels) and chlorophyll autofluorescence (red, centre-left panels), as well as under brightfield illumination (right panels). An overlay of the YFP and chlorophyll images is presented (centre-right panels). Similar analyses of an equivalent series of three atTic22-III YFP constructs produced identical results (data not shown). Scale bar = 10 µm. B, Analysis of the localization of the Arabidopsis atTic22-III protein following in vitro import. Chloroplasts isolated from 14-day-old wild-type plants were used in protein import assays with either [35S]-methionine-labelled atTic22-III or similarly-labelled Rubisco small subunit (SSU) precursor as a control. All import reactions were allowed to proceed for 20 minutes. At the end of the import reactions, the chloroplasts were recovered and treated in the absence (−) or presence (+) of either thermolysin or trypsin, prior to analysis by SDS-PAGE and fluorography. TM indicates an aliquot of the atTic22-III or SSU translation mixture equivalent to 10% of the amount added to each assay; p and m indicate the precursor and mature forms of SSU, respectively. The mature from of Rubisco SSU is located in the stroma, and so is expected to be resistant to both proteases; on the other hand, un-imported SSU precursor is expected to be sensitive to both proteases. Concerning atTic22-III, resistance to thermolysin and sensitivity to trypsin is consistent with localization to the intermembrane space. (TIF) [file pone.0063863.s002.tif]

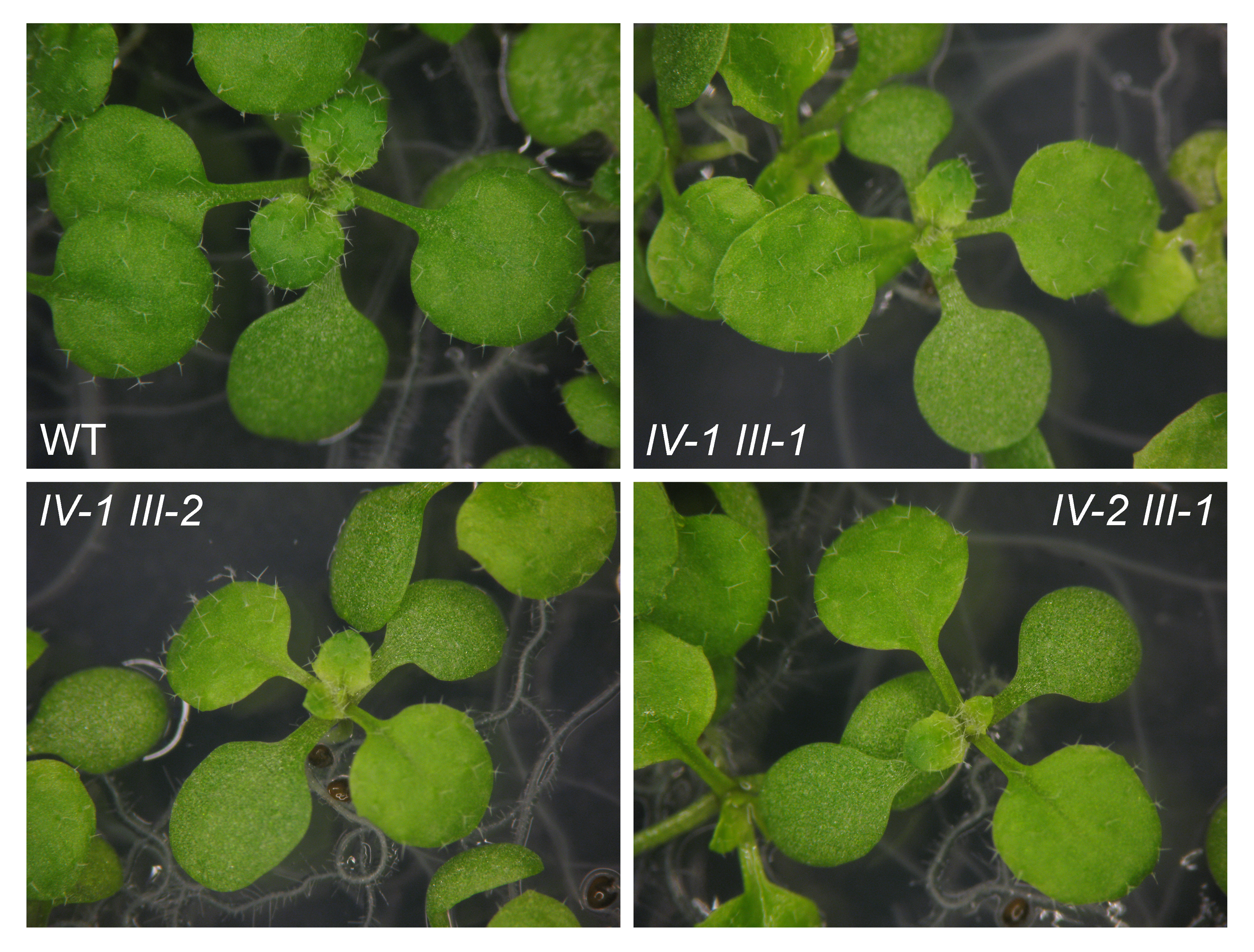

Supplement: Figure S3 — Visible appearance of 14-day-old tic22 double-mutant plants. Homozygous plants of the indicated tic22 double-mutant genotypes were grown alongside wild type in vitro for 14 days. Representative plants were then photographed. The images illustrate clearly that the chlorosis associated with loss of Tic22 is not restricted to the cotyledons, and can also be seen in true leaves. (TIF) [file pone.0063863.s003.tif]
